# Efficacious patient‐specific QA for Vertebra SBRT using a high‐resolution detector array SRS MapCHECK: AAPM TG‐218 analysis

**DOI:** 10.1002/acm2.14276

**Published:** 2024-02-27

**Authors:** Kemal Berk, Tomas Kron, Nicholas Hardcastle, Adam Unjin Yeo

**Affiliations:** ^1^ Department of Physical Sciences Peter MacCallum Cancer Centre Melbourne Victoria Australia; ^2^ Sir Peter MacCallum Department of Oncology the University of Melbourne Melbourne Victoria Australia; ^3^ Centre for Medical Radiation Physics University of Wollongong Wollongong NSW Australia

**Keywords:** patient‐specific quality assurance (PSQA), SRS MapCHECK, TG‐218, vertebra SBRT

## Abstract

**Purpose:**

Patient‐specific quality assurance (PSQA) for vertebra stereotactic body radiation therapy (SBRT) presents challenges due to highly modulated small fields with high‐dose gradients between the target and spinal cord. This study aims to explore the use of the SRS MapCHECK® (SRSMC) for vertebra SBRT PSQA.

**Methods:**

Twenty vertebra SBRT treatment plans including prescriptions 20 Gy/1 fraction and 24 Gy/2 fractions were selected for each of Millennium (M)‐Multileaf Collimator (MLC), and high‐definition (HD)‐MLC. All 40 plans were measured using Gafchromic EBT3 film (film) and SRSMC, using the StereoPHAN phantom. Plan complexity was assessed using modulation complexity score (MCS), edge metric (EM) (mm^−1^), modulation factor (MU/cGy), and average leaf pair opening (ALPO) (mm) and its correlation with gamma‐pass rate was investigated. The high dose gradient between the target and the spinal cord was analyzed for film and SRSMC and compared against the treatment planning system (TPS). Applying the methodology proposed by AAPM TG‐218, action and tolerance values specific to the SRSMC for vertebra SBRT were determined for β values ranging from 5 to 8.

**Results:**

Film and SRSMC gamma‐pass rates showed no correlation (*p* > 0.05). A moderate negative correlation (*R* = ‐0.57, *p* = 0.01) is present between EM and SRSMC 3%/1 mm gamma‐pass rate for HD‐MLC plans. Both film and SRSMC accurately measured high dose gradients between the target and the spinal cord (*R* ^2^ > 0.86, *p* ≤ 0.05). Notably, dose‐gradient of HD‐MLC plans is 22% steeper and has a smaller standard deviation to M‐MLC plans (*p* ≤ 0.05). Applying TG‐218, the film tolerance limit was 96% with action limit 95% for 5%/1 mm (β = 6) and for the SRSMC tolerance limit was 97% with an action limit of 96% for 4%/1 mm (β = 6).

**Conclusion:**

Our findings suggest that universal TG‐218 limits may not be suitable for vertebra SBRT PSQA. This study demonstrates that SRSMC is a viable tool for vertebra SBRT PSQA, supported by TG‐218 implementation of process‐based tolerance and action limits.

## 1 INTRODUCTION

Stereotactic body radiation therapy (SBRT) is used to treat vertebra metastases for both pain relief and control of metastatic disease.^1^, ^2^ When using SBRT for vertebra metastases, it is critical to minimize time between treatment simulation and treatment. Dosimetry in vertebra SBRT may have increased uncertainty due to the substantial modulation required to deliver a high tumor dose while meeting constraints of immediately adjacent structures such as the spinal cord and esophagus. While modulation can help spare these critical structures, it can also increase the uncertainty in the dose delivery.^3^ Vertebra SBRT thus requires stringent patient‐specific quality assurance (PSQA), which may be challenging given the time constraints associated with time‐sensitive treatment for pain control.

For vertebra SBRT planning, the area around the spinal cord, which is usually the dose‐limiting structure, needs to have a very conformal and sharp dose falloff. Therefore, a high‐resolution detector is desired for PSQA of vertebra SBRT plans to verify the dose gradient between the target and the spinal cord can be delivered as planned. Due to the small dimensions and steep high dose gradients of photon beams used for SBRT‐type treatments, TG 101 recommends using dosimeters with a spatial resolution of approximately 1 mm or better to measure basic dosimetry data along with detailed dose correction factors applied.^4^ Film is routinely used for SBRT PSQA due to its high spatial resolution and ability to be placed in specific anatomical planes, despite its limitations associated with time‐consuming and labor‐intensive processes as well as a high degree of uncertainty due to intra‐batch variations. This is also relevant to accredited dosimetry audits as well as a trial credentialling process.^5^, ^6^

High‐resolution solid‐state arrays have been introduced primarily for intracranial stereotactic radiotherapy quality assurance. Such arrays are potentially attractive for vertebra SBRT as they have the potential to facilitate rapid, high‐resolution PSQA. While there are numerous publications on the use of the SRS MapCHECK® (Sun Nuclear, Melbourne) (SRSMC) for stereotactic radiosurgery (SRS) intracranial small field PSQA verification, there is currently no literature on the use of SRSMC to establish tolerance and action limits specifically for vertebra SBRT PSQA verification,^7^, ^8^ which possess the aforementioned distinct features compared to other plans. The AAPM TG‐218 report covers patient‐specific intensity modulated radiation therapy (IMRT) quality assurance (QA) in great detail and offers suggestions for methods for customized tolerance limits.^9^

This study aims to compare the SRSMC with film for PSQA of vertebra SBRT treatment plans, including the verification of the high dose gradient between the target and the spinal cord. The study also aims to establish tolerance limits and action limits for SRSMC and film for vertebra SBRT purposes, using the guidelines outlined for process‐based action and tolerance limits in TG218.^9^

## 2 MATERIALS AND METHODS

### 2.1 Plan selection

The treatment planning cohorts consisted of two separate groups with two different prescriptions. Set‐A contained ten plans with the same target geometry and with varying levels of complexity from the 2017 TROG planning challenge.^10^ The thoracic vertebra target in this plan had a prescription of 20 Gy in 1 fraction (fx) as per the plan challenge instruction. Set‐B consisted of ten plans with ten differing target geometry and complexity for patients treated in our institution. The targets were three cervical vertebra, four thoracic vertebra and three lumbar vertebra with a prescription of 24 Gy in 2 fractions as per our department protocol. Planning goals for Set‐A were GTV (D99%), PTV (D90%) to receive 20 Gy, PTV D0.03cc to 30−32.5 Gy, and dose limits for spinal cord (D0.03cc < 12 Gy), esophagus (D0.03cc < 15.4 Gy) and cauda equine (D0.03cc < 16 Gy). Planning goals for Set‐B were GTV (D99%), PTV (D90%) to receive 24 Gy, PTV D0.03cc to be 25–27 Gy, and dose limits for spinal cord (D0.03 < 14 Gy) and esophagus (D0.03cc < 20 Gy). Note that dose limits for spinal cord are approximately 60% for both prescriptions.

### 2.2 Calculation model and plan complexity

For each plan, two models of linear accelerators were studied: the Varian TrueBeam with the Millennium (M) multileaf collimator (MLC) system and the Varian TrueBeam STx with the High Definition (HD) MLC system. The 40 central leaves of the M‐MLC system were 5 mm wide, while the 20 outer leaves were 10 mm wide, whereas the central 32 leaves of the HD‐MLC system were 2.5 mm wide, and the outer 28 leaves were 5 mm wide.

All treatment plans were generated by using volumetric modulated arc therapy (VMAT) technique in Eclipse treatment planning system (TPS) (Varian Medical Systems, Palo Alto) with AcurosXB algorithm (v15.6.06) reporting dose to medium for dose calculation and Photon Optimization algorithm (v15.6.06) for optimization. Dose grid size was 1.25 mm as per our departmental protocol for SRS/SBRT plans.

The following complexity metrics were computed for all treatment plans: Modulation Complexity Score (MCS), Edge Metric (EM), Monitor units per Grey (MU/cGy), and Average Leaf Pair Opening (ALPO)^11^, ^12^ MCS measures the variability in shape of MLC segments, while EM uses MLC y‐leaf side length normalized by aperture area. MU/cGy is the number of monitor units required to deliver one Gray of dose, and ALPO is a measure of how closed the MLC‐defined field is.

### 2.3 Measurement detectors

To calibrate the film, twelve pieces were irradiated using Varian TrueBeam 10 megavoltage (MV) beam with a dose rate of 600 MU/min. Solid water served as the phantom material. The irradiation took place at a 100 cm source‐to‐surface distance, with a 10 × 10 cm^2^ field size. The film was positioned at the location of maximum dose (Dmax), maintaining conditions of maximum backscatter. Film was irradiated in doses from 0 Gy up to 30 Gy.

Film's resolution as a detector is defined by the resolution of the scanned image. Film was scanned using an Epson V700 scanner (Seiko Epson Corporation, Nagano, Japan) in 48 bits with 75 dpi resolution and with no color correction, resulting in its resolution of 0.35 mm.

The SNC Patient Software (v8.4.0, Sun Nuclear Corporation, Melbourne, FL) film analysis tool, along with green channel analysis 13, was utilized for both the creation of the film calibration curve and the gamma analysis of the film dose distribution.^13^

The SRSMC contained 1013 n‐type solid state diodes in a 7.7 × 7.7 cm^2^ array with a 2.47 mm diagonal center‐to‐center spacing as a detector resolution. The polymethyl methacrylate (PMMA) housing measures 320 mm^3^ × 105 mm^3^ × 45 mm^3^ and contains the detector array. The array's diodes have an active size of 0.48 mm × 0.48 mm which complies with TG‐101^4^ standards. The SNC patient software takes into account over‐response at small field sizes (20 mm × 20 mm or less) as well as temperature, pulse repetition rate, and angular dependencies that are typical of diodes.^7^

The SRSMC was calibrated for each energy, according to the instructions set by the manufacturer. The array calibration systematically evaluates the relative sensitivity differences among detectors, effectively mitigating response discrepancies and ensuring uniformity across the array. The angular corrections for the SRSMC are energy‐dependent, therefore an array calibration per beam energy is recommended. A dose calibration was conducted for each measurement session, following the manufacturer's instructions. The calibration process involved delivering a known dose to the SRSMC, positioned within the StereoPHAN at the isocentre of the linac. Subsequently, the central diode dose was input into the software, facilitating the establishment of the absolute dose calibration for the array.

### 2.4 Measurement and analysis

The SRSMC and film were separately inserted into the StereoPHAN (Sun Nuclear Corporation Melbourne, FL) sagittal plane to measure all treatment plans. Spatial registration was performed via image‐guided radiotherapy (IGRT). SNC Patient software was used to compare the measured film and SRSMC 2D dose map to the TPS exported dose map using gamma analysis (Γ) with 3%/1 mm (dose‐difference/distance‐to‐agreement [DTA]), 4%/1 mm and 5%/1 mm dose difference and DTA criteria in absolute dose mode, and a 10% dose threshold. In accordance with the TG‐218 recommendation,^9^ the dose‐difference criteria of 3% to 5% are clinically acceptable dose difference limits. To ensure the measurements are sensitive to errors associated with positioning of the high dose gradient region, the chosen DTA value remained fixed at 1 mm.

**FIGURE 1:**
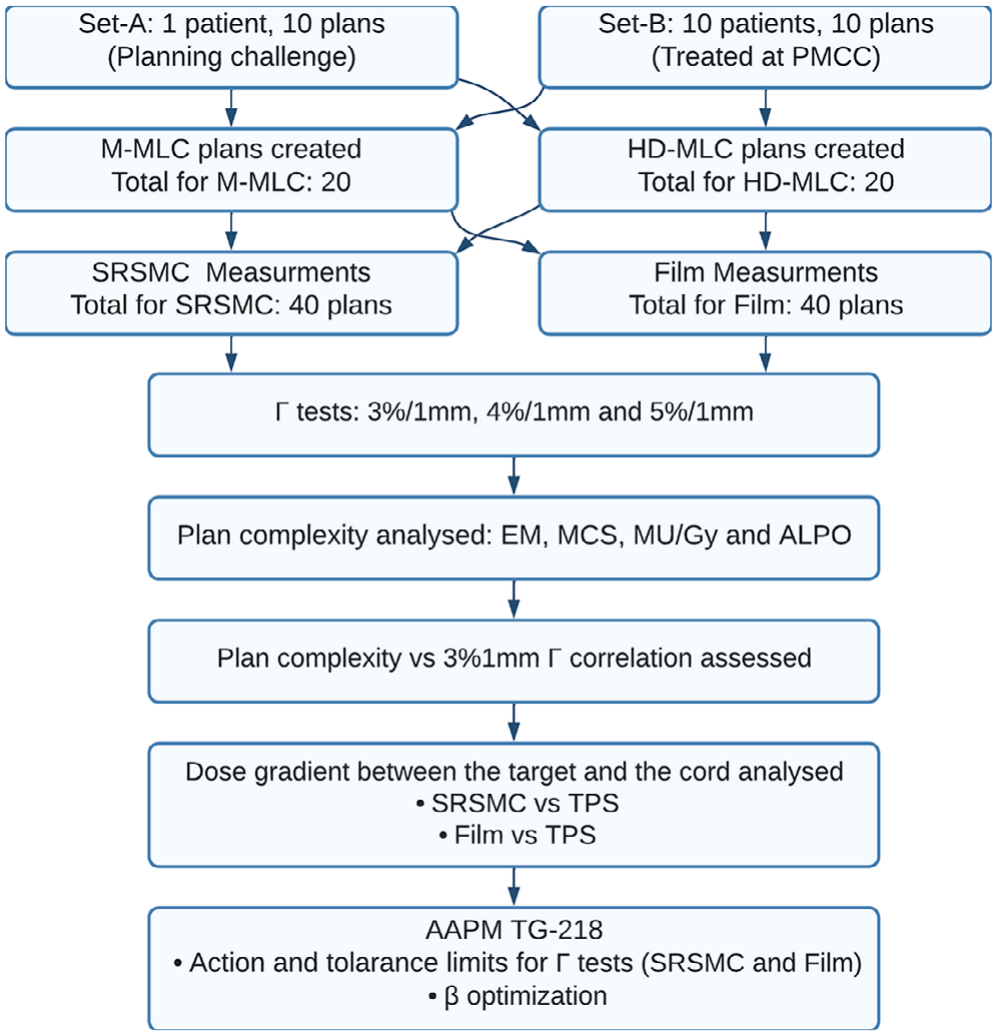
Example of the location the dose profile was obtained from TPS, Film and SRSMC for high dose gradient analysis.

The dose gradient between the target and the spinal cord analysis is performed in terms of total prescription dose, for Set A measurements this equates to 20 Gy in 1 fraction and for Set B 24 Gy in 2 fractions. The dose gradient between the target and the spinal cord is quantified from the anterior‐posterior line profile through the center of the target on the measured sagittal plane (see Figure 1 as an example). This is inevitably defined as the highest measurable dose‐gradient in SRSMC measurements (which has the coarsest grid resolution of 2.47 mm) using two measurement points within the dose gradient between the target and the spinal cord. The corresponding dose points from film measurements and TPS calculation were used to define respective dose‐gradients at the same points from SRSMC. The highest measurable dose‐gradient from SRSMC always lies on a linear part of dose gradient shown in film and TPS including spinal cord dose limit (which is about 60% of prescription dose); thus, a good surrogate to quantify dose‐gradient between the target and the spinal cord. The dose gradient of SRSMC and film was compared with TPS to ensure the measurement device resolution is capable of measuring the planned dose distribution.

### 2.5 TG 218 application

Statistical process control can be utilized to determine action limits from patient‐specific QA measurements when universal action limits are inappropriate. TG 218 explains this procedure in further detail.^9^ We applied the process presented in the TG 218 section 9 to vertebra SBRT treatment PSQA. In particular, the action limits were calculated with

(1) $$\Delta A\;=\;\beta \sqrt{\sigma^{2}+{\left(\bar{x}-T\right)}^{2}}\;$$

where ΔA represents the difference between the upper and lower action boundaries, T is the process goal value, and $\sigma^{2}$ and $\bar{x}$ are the process variance and process mean, respectively. The constant β in this approach is a combination of two factors. One factor originates from the process capability metric as a cutoff for an acceptably performing process. The other factor balances type I errors (rejecting the null hypothesis when it is true) and type II errors (not rejecting the null hypothesis when it is false) when using PSQA measurements to make a decision about process performance.

Since the gamma analysis performed comparing SRSMC and film‐measured dose to TPS has a defined upper limit of 100%, the lower control limit was employed as the tolerance limit. The lower control limit was calculated using the statistical control procedure detailed in TG‐218 Section 8,^9^

(2) $$centre\;line\;=\;\frac{1}{n}\sum_{1}^{n}x$$

(3) $$lower\;control\;limit\;=\;centre\;line-2.66\cdot \;\bar{mR}$$

(4) $$\bar{mR}=\frac{1}{n-1}\;\sum_{i\;=\;2}^{n}\left|x_{i}-x_{i-1}\right|$$

where n is the number of measurements performed, *x* is the individual PSQA measurement and $\bar{mR}$ is the moving range.

All following results combine data from both prescription sets (Set‐A and Set‐B), ensuring a like‐for‐like comparison without differentiating analysis based on plan prescription. Results obtained for Film and SRSMC for each M‐MLC and HD‐MLC plan are differentiated on all following results, in order to assess the impact of the measurement device and MLC system.

### 2.6 Statistical analysis

We utilized Spearman correlation analysis to investigate the relationship between the gamma pass rate (Γ) obtained from both film and SRSMC measurements. Additionally, we employed this analysis to assess the correlation between complexity metrics and the Γ results. Furthermore, we evaluated the dose gradient scores obtained from both film and SRSMC and compared them to the TPS using a regression analysis. Statistical significance was defined as results with a *p*‐value of ≤ 0.05.^14^ To compare the TPS dose gradient scores between M‐MLC and HD‐MLC, we first assessed the normal distribution of the data using the Shapiro‐Wilk test.^14^, ^15^ Subsequently, a paired t‐test was conducted to compare these dose gradient scores. Once again, statistical significance was defined as results with a *p*‐value of ≤ 0.05.

## 3 RESULTS

### 3.1 Film and SRSMC measurements

**FIGURE 2:**
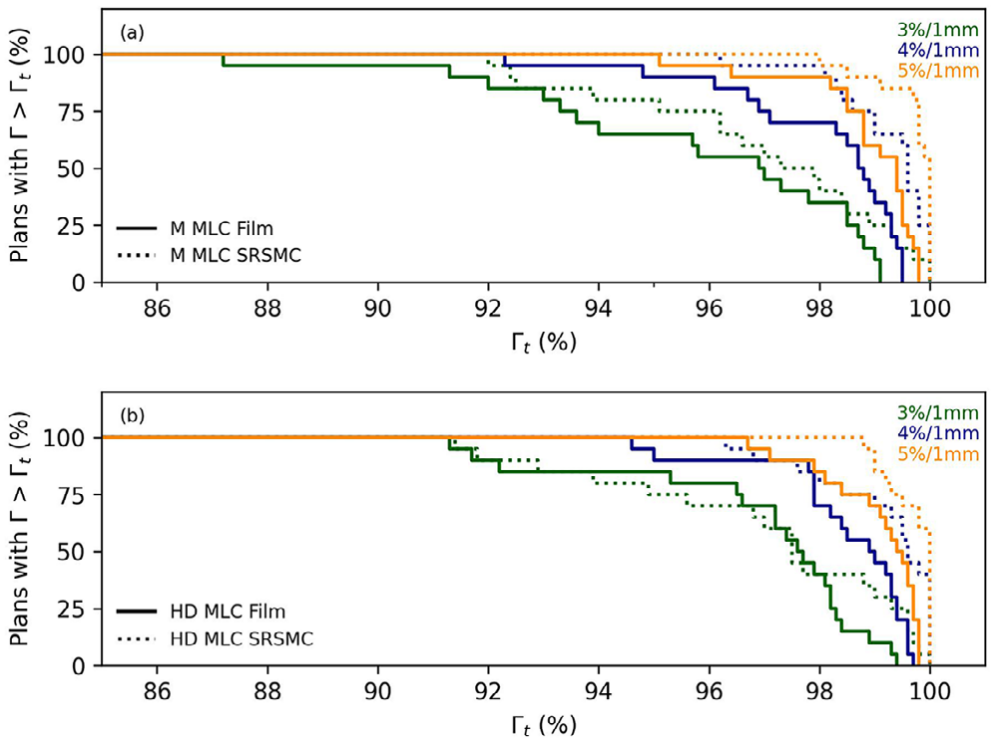
Plot showing the percentage of plans with gamma pass rates (Γ) greater than the gamma pass rate threshold (Γt) for (a) M‐MLC (top) and (b) HD‐MLC (bottom) groups, as determined by film and SRSMC. The legend indicates the gamma pass rate tolerances.

Figure 2 depicts the percentage of plans that surpass the gamma pass rate (Γt > Γ) for M‐MLC Film Γ and SRSMC Γ results and HD‐MLC Film Γ and SRSMC Γ results using gamma criteria of 3%/1 mm, 4%/1 mm, and 5%/1 mm. For M‐MLC Film, the gamma pass rates ranged from 87.2 to 99.1, 92.3 to 99.5, and 95.1 to 99.8 for the respective gamma criteria. The SRSMC M‐MLC results demonstrated gamma pass rates ranging from 92.0 to 100.0, 96.2 to 100.0, and 98.0 to 100.0. Moving to HD‐MLC Film, the gamma pass rates were observed to range from 91.3 to 99.4, 94.6 to 99.7, and 96.7 to 99.8. Finally, for HD‐MLC SRSMC, the gamma pass rates were found to range from 91.4 to 100.0, 96.3 to 100.0, and 98.8 to 100.0. The M‐MLC samples consistently displayed slightly lower gamma values compared to their HD‐MLC counterparts at corresponding percentages. In general gamma analysis showed that SRSMC achieved higher Γ compared to film, except Γ range between 94% and 98% for the HD‐MLC with 3%/1 mm gamma criteria, which shows similar results for the two detectors film and SRSMC.

**FIGURE 3:**
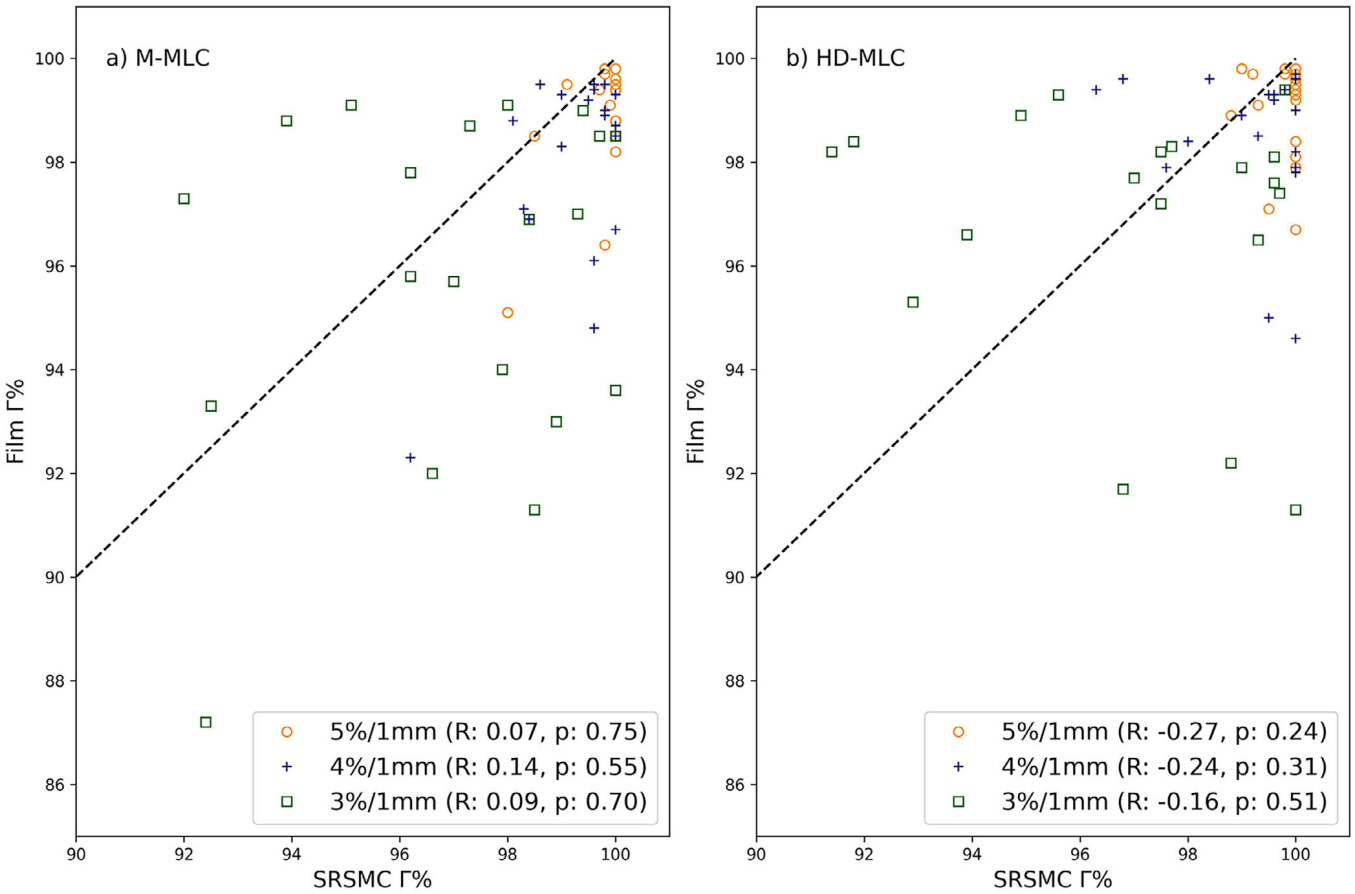
Film Gamma pass rate versus SRSMC gamma pass rate for (a) MMLC and (b) HD‐MLC plans, with the dotted line representing the line of identity. Spearman correlation analysis indicated no correlation between film gamma pass rates and SRSMC gamma pass rates (*p* > 0.05) for all gamma criteria and for either M‐MLC or HD‐MLC plans.

Figure 3 illustrates the relationship between film and SRSMC gamma pass rates for each individual plan measured. The black dotted line in both figures represents the line of identity. Spearman correlation analysis was conducted separately for M‐MLC and HD‐MLC plans to assess the predictive capability of film results on SRSMC results and vice versa.

M‐MLC plans exhibited Spearman correlation coefficients (R) of 0.09, 0.14, and 0.07 for 3%/1 mm, 4%/1 mm, and 5%/1 mm criteria, respectively, with corresponding *p*‐values of 0.7, 0.55, and 0.75. For HD‐MLC plans, the correlation coefficients were −0.16, −0.25, and −0.27 for 3%/1 mm, 4%/1 mm, and 5%/1 mm criteria, respectively, with *p*‐values of 0.51, 0.31, and 0.24. Notably, the results indicated no significant correlation between film gamma pass rates and SRSMC gamma pass rates for either M‐MLC or HD‐MLC plans.

### 3.2 Correlation of complexity metrics with Γ

**FIGURE 4:**
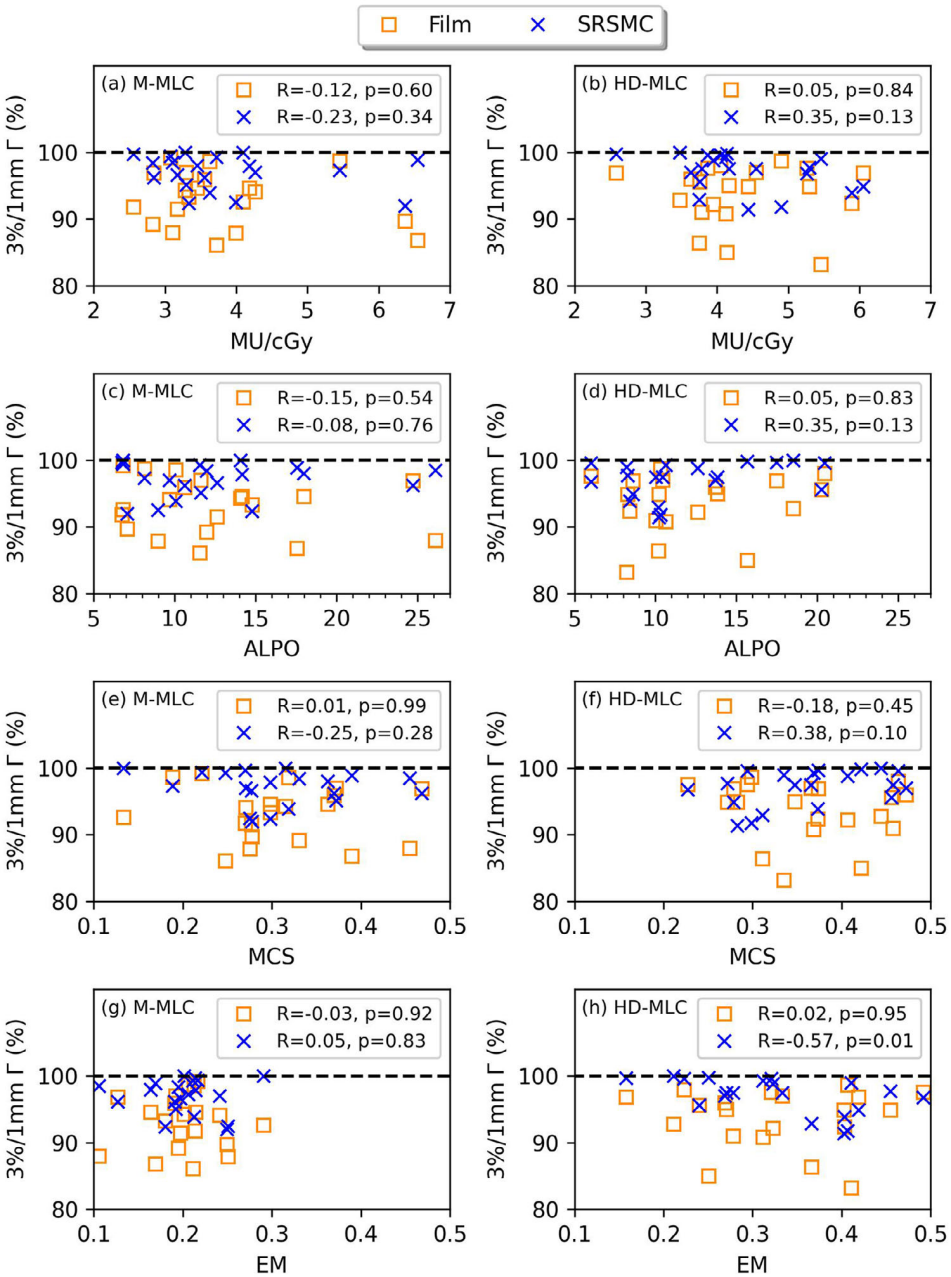
SRSMC and Film 3%/1 mm gamma pass rate values plotted against complexity metrics (a) MU/cGy (M‐MLC), (b) MU/cGy (HD‐MLC), (c) ALPO (M‐MLC), (d) ALPO (HD‐MLC), (e) MCS (M‐MLC), (f) MCS (HD‐MLC, (g) EM (M‐MLC) and (h) EM (HD‐MLC). No correlation between plan complexity and gamma pass rate for all comparison analyses, except for SRSMC gamma pass rate against EM for the HD‐MLC in figure (h).

In Figure 4 subplots (a) through (h), each representing a specific complexity metric, we observe Spearman correlation between the SRSMC and Film 3%/1 mm gamma pass rates. (a) MU/Gy (M‐MLC) for Film *R* = −0.12, *p* = 0.6; for SRSMC: *R* = −0.23, *p* = 0.34 (b) MU/Gy (HD‐MLC) for Film: *R* = 0.05, *p* = 0.84; for SRSMC *R* = 0.35, *p* = 0.13 (c) ALPO (M‐MLC) for Film: *R* = −0.15, *p* = 0.54; for SRSMC *R* = −0.08, *p* = 0.76, (d) ALPO (HD‐MLC) for Film *R* = 0.05, *p* = 0.13; for SRSMC *R* = 0.35, *p* = 0.13, (e) MCS (M‐MLC) for Film *R* = 0.01, *p* = 0.99; for SRSMC *R* = −0.25, *p* = 0.28, (f) MCS (HD‐MLC) for Film: *R* = −0.18, *p* = 0.45; for SRSMC: *R* = −0.18, *p* = 0.10, (g) EM (M‐MLC) for Film: *R* = −0.03, *p* = 0.92; for SRSMC *R* = 0.05, *p* = 0.83, (h) EM (HD‐MLC) for Film = R 0.02, *p* = 0.95; for SRSMC *R* = −0.57, *p* = 0.01. Notably, only the correlation between SRSMC and Film in HD‐MLC plans using the EM complexity metric shows a a negative moderate correlation (R −0.57, *p* = 0.01) which is statically significant (*p*‐value of ≤ 0.05).

### 3.3 Dose gradient analysis

**FIGURE 5:**
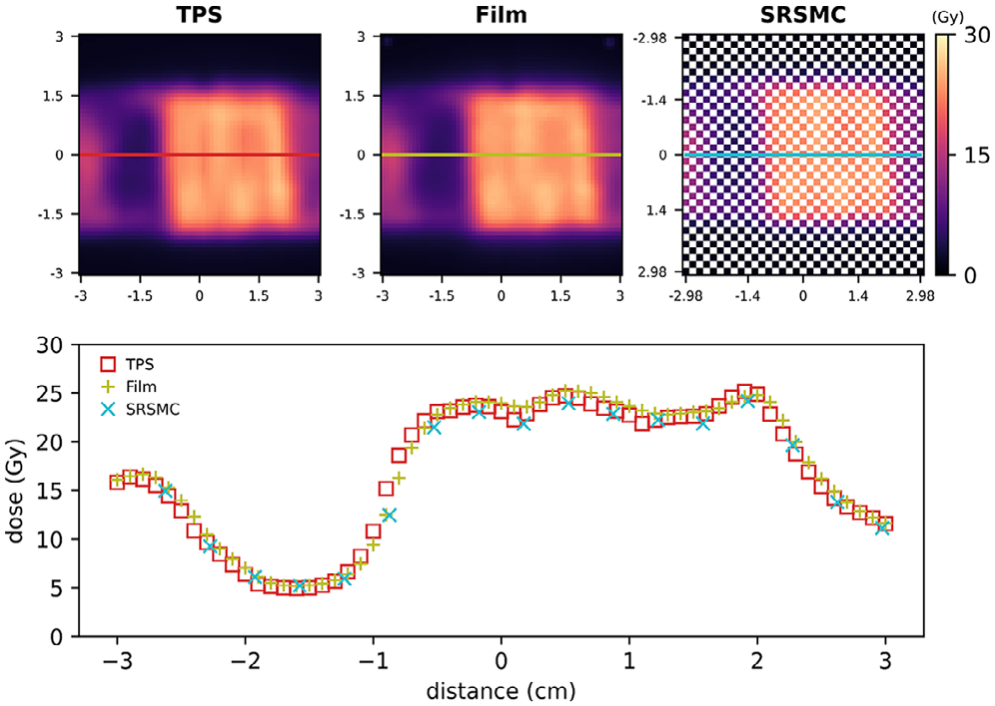
High dose gradient regression analysis for (a) M‐MLC Film versus TPS, (b) M‐MLC SRSMC versus TPS, (c) HD‐MLC Film versus TPS, and (d) HD‐MLC SRSMC versus TPS. Units in cGy/mm.

Figure 5 displays the dose profile of the SRSMC, Film, and TPS along the posterior‐to‐anterior direction between the target and the spinal cord. The detector resolution for SRSMC is 2.47 mm diagonal center‐to‐center separation; film, on the other hand, has a resolution of 0.35 mm while the dose‐grid size for TPS is 1.25 mm.

**FIGURE 6:**
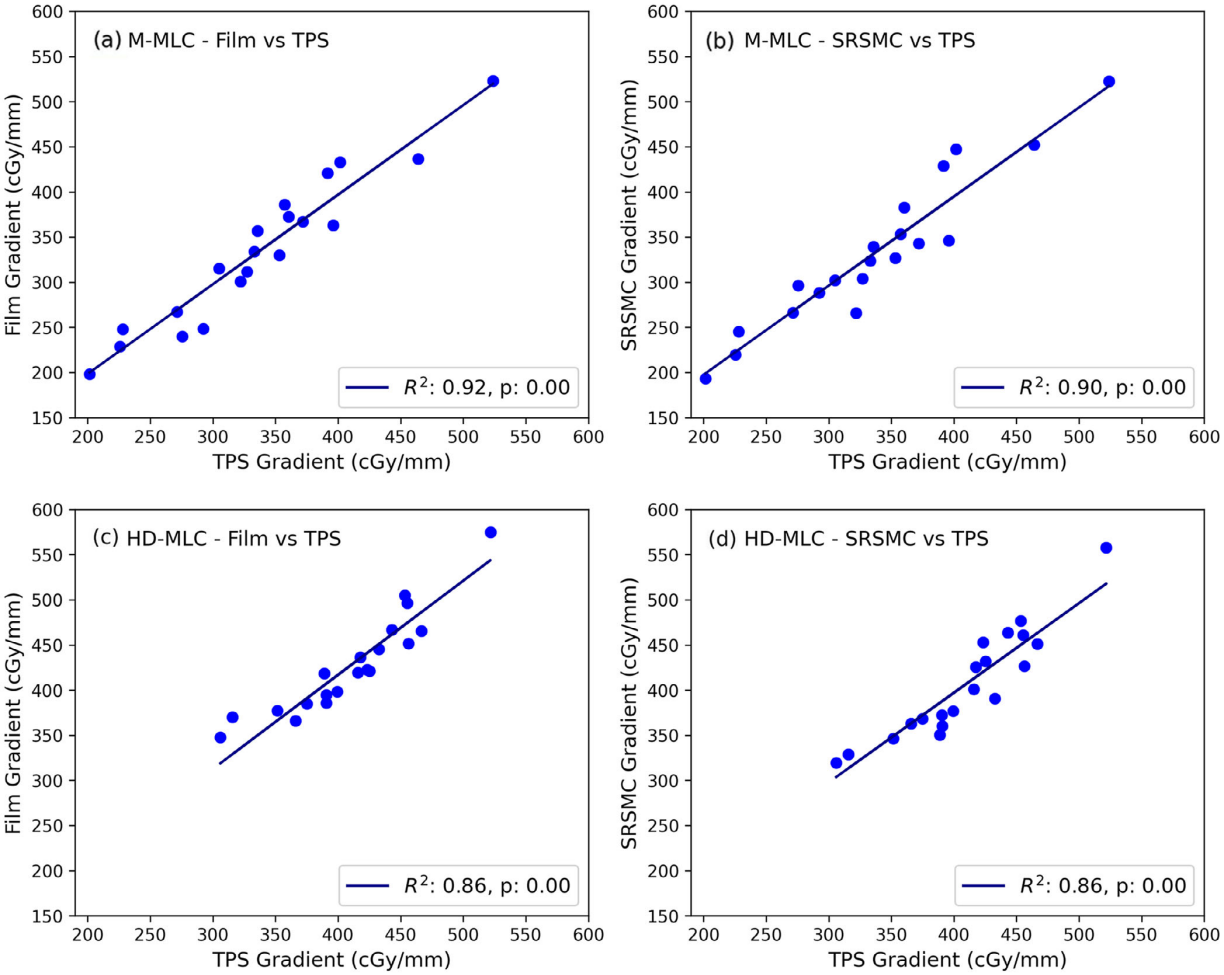
Summary of the workflow used in this study.

Figure 6 illustrates the regression analysis conducted to assess the high dose gradient measured by both the SRSMC and film, in comparison to the planned TPS values. Subplots (a) M‐MLC Film versus TPS has *R* ^2^ = 0.92 with *p* = 0.00, (b) M‐MLC SRSMC versus TPS has *R* ^2^ = 0.90 with *p* = 0.00, c) HD‐MLC Film versus TPS has *R* ^2^ = 0.86 with *p* = 0.00 and d) HD‐MLC SRSMC versus TPS has *R* ^2^ = 0.86 with *p* = 0.00. In general, both detectors were able to resolve the high dose gradient observed in the TPS dose calculation, with *p*‐values below 0.05, indicating the linear regression values are statistically significant. Since both detectors can measure the dose gradient between the target and the spinal cord, the following comparison is for TPS values only.

In the M‐MLC scenario, the mean TPS gradient stands at 336.83 and a standard deviation of 76.87. In contrast, the HD‐MLC scenario displays a 22% higher mean TPS gradient of 409.71, accompanied by a narrower spread indicated by a standard deviation of 50.75. The paired t‐statistic of −3.880 and a *p*‐value of 0.001 further highlight the difference is statistically significant (*p*‐value of ≤ 0.05).

### 3.4 TG‐218 application

Table 1 shows the summary of process‐based tolerance and action limits based on TG‐218 application^9^ based on gamma pass rate results for both detectors from Figure 4. Gamma passing‐rate action limit was calculated using Equation (1) and tolerance limit values were calculated using Equations (2) and (3) for the most commonly used passing criteria, such as 3%/1 mm, 4%/1 mm and 5%/1 mm with β values from 5 to 8 for two difference MLC types (M‐MLC and HD‐MLC) measured by Film and SRSMC. As per definition, the tolerance level must be greater than the action level. A change in the value of β signifies a change in the performance threshold for an acceptable procedure. In general, the SRSMC results show a tighter variation of tolerance limits (ranging 90−99%) across all gamma passing criteria for both MLC types, compared to the film results (ranging 81%−96%). The same trend holds for variations of action limits across β values from 5 to 8 for both MLC types, that is, ranging from 84% to 99% for SRSMC, compared to its range 68%−95% for film across the three gamma passing criteria.

**TABLE 1:** Action and tolerance values for M‐MLC and HD‐MLC, B 5−8 for gamma values 3−5%/1.

				Action limit (%)			
MLC system	Measurement device	Gamma criteria	Tolerance limit (%)	β = 5	β = 6	β = 7	β = 8
HD‐MLC	Film	5%/1 mm	96	95	95	94	93
		4%/1 mm	93	91	89	87	85
		3%/1 mm	84	81	77	73	69
M‐MLC	Film	5%/1 mm	95	95	94	93	92
		4%/1 mm	90	90	89	87	85
		3%/1 mm	81	80	76	72	68
HD‐MLC	SRSMC	5%/1 mm	99	99	99	98	98
		4%/1 mm	97	97	96	95	94
		3%/1 mm	90	90	88	86	84
M‐MLC	SRSMC	5%/1 mm	99	99	98	98	98
		4%/1 mm	97	97	96	96	95
		3%/1 mm	91	90	88	86	84

## 4 DISCUSSION

This study has compared the SRSMC to Gafchromic EBT3 film for 2 types of MLC, the M‐MLC and HD‐MLC with the aim of determining if the suitability of the SRSMC for vertebra SBRT PSQA. Figure 3 shows the relationship between gamma pass rate (Γ) for film and SRSMC for both (a) M‐MLC and (b) HD‐MLC at different gamma criteria levels (3%/1 mm, 4%/1 mm, and 5%/1 mm). Spearman correlation results for Film Γ versus SRSMC Γ for 3%/1 mm, 4%/1 mm, and 5%/1 mm do not correlate. A high/low Γ measurement on one device can't be used as an indication for the measurement outcome on the other device.

The design of our study is carefully controlled to investigate correlation of PSQA results with plan complexity as the analysis focuses on a single body site, that is, vertebra SBRT for a given TPS model per MLC type. The study used multiple plan complexity metrics MCS, EM, MU/cGy and ALPO for the M‐MLC and HD‐MLC machines, to perform a spearman correlation analysis between complexity metrics and Γ. Only 3%/1 mm gamma criteria was used for correlation analyses because more forgiving criteria with higher dose levels have Γ values near 100% as seen in Figure 2, which impacts correlation scores. A moderate negative correlation is present for EM versus Γ (*R* = −0.57, *p* = 0.01) for the SRSMC measurement performed on the HD‐MLC system. Higher EM values are associated with more modulated plans, which is correlated with a lower gamma pass rate of SRSMC measurement for HD‐MLC plans. The total range of EM investigated for HD‐MLC (0.16‐0.49) was wider compared to total range of M‐MLC (0.11‐0.29). This may be a factor of why correlation of EM versus Γ only being apparent for HD‐MLC, and not for M‐MLC.

This finding is in line with the study performed by Rose et al.^8^ that investigated SBRT plans for different body sites. Despite the study design not looking at correlation between plan complexity and gamma pass rates because of the nature of their multi‐institutional study using a mix of TPS types and models, they have highlighted the importance of exercising caution when delivering highly modulated plans to the SRSMC. It is crucial to carefully differentiate errors arising from TPS modelling and those related to the detector to ensure accurate assessment of SABR vertebra PSQA results.

In the assessment of SABR vertebra PSQA, accurately evaluating the gradient and positioning of the high dose gradient between the target and the spinal cord is crucial due to the spinal cord's role as the dose limiting structure. This evaluation is essential alongside the gamma pass rate in SABR vertebra PSQA. The use of 1 mm DTA is to ensure the gamma analysis is sensitive to positional errors. To distinguish between positional errors that arise from measurement device location and dose delivery positional errors, IGRT is used. Figure 5 displays the results for the high dose gradient score obtained using film and the SRSMC, compared to the values from the TPS. Regression analysis reveals high R‐squared values, indicating that both measurement devices have sufficient resolution to accurately capture the high dose gradient between the target and the spinal cord. This provides confidence that the SRSMC is as capable as film when assessing the dose gradient for vertebra SBRT (See Figure 6).

In the analysis of Table 2, it is noteworthy that the dose gradient between the target and the spinal cord for HD‐MLC plans are 22% steeper compared to M‐MLC plans. This finding is statistically significant with a *p*‐value of ≤ 0.05. Additionally, the data indicates greater consistency in HD‐MLC plans, as evidenced by the smaller standard deviation compared to M‐MLC plans.

**TABLE 2:** Comparison of mean TPS gradient and standard deviation in M‐MLC and HD‐MLC scenarios.

MLC system	Mean TPS gradient	Standard deviation
**M‐MLC**	336.83	76.87
**HD‐MLC**	409.71	50.75

In Table 1, we demonstrate the application of TG‐218^9^ by presenting the gamma pass rate action and tolerance values for 3%/1 mm, 4%/1 mm, and 5%/1 mm criteria, considering β values ranging from 5 to 8. The measurements were conducted using two different MLC types, namely M‐MLC and HD‐MLC, with assessments performed using Film and SRSMC methods. The selection of β values is crucial in determining the sensitivity of process monitoring. A lower β value indicates a more sensitive approach capable of detecting smaller discrepancies, while a higher β value reduces the likelihood of unnecessary process failures. To ensure that the action limit does not exceed the tolerance limit, we set the minimum value of β investigated at 5. This selection approach ensures that the chosen tolerance and action values align with the desired criteria for accurate assessment of γ results, striking a balance between sensitivity and minimizing false negative results.

It is important to note that film and SRSMC should have separate tolerance values, as film gamma results consistently differed from SRSMC results, as shown in Figure 2. Furthermore, Figure 3 demonstrated that the gamma pass rate of SRSMC results does not correlate consistently with film results. Choosing appropriate action and tolerance limits is crucial for safe and effective treatment. TG‐218 does not recommend specific action and tolerance limits for SBRT treatments. TG‐218 suggests universal tolerance criteria of ≥95% with a 3%/2 mm and a 10% dose threshold, and action limits of ≥90% with the same gamma criteria. The gamma criteria and action and tolerance limits calculated in this study are more stringent than TG‐218′s recommendations. In vertebra SBRT, a 1 mm DTA is important for assessing the precise positioning of the high dose gradient region. Dose criteria of 3%, 4%, and 5% were included in our analysis as a higher discrepancy in dose is less clinically relevant for SBRT treatments when compared to positional discrepancies. This approach provides enhanced robustness in addressing positional errors while simultaneously allowing flexibility for dose discrepancies.

Our clinical experience suggests that evaluating multiple gamma criteria in addition to gamma analysis may be beneficial in gaining insights into potential factors contributing to measurement errors. The dose gradient, and determination the spinal cord is appropriately spared by the tolerance isodose line are just as critical in vertebra SBRT PSQA.

The study has some inherent limitations. Given that all measurements are conducted using the StereoPHAN, the feasibility of obtaining axial plane measurements is restricted, and only sagittal plane measurements are viable. This limitation might compromise the ability to capture information valuable for certain dose distributions, particularly those enveloping the spinal cord from various angles.

Additionally, the confined measurement area, limited to 7.7 × 7.7 cm^2^ for both the SRSMC and film when utilized in the StereoPHAN, poses a constraint. Consequently, some vertebra lesions that exceed this measurement area may be challenging to effectively assess. To address this limitation, the incorporation of a secondary phantom designed for axial measurements and capable of accommodating larger lesions would be beneficial alongside the StereoPHAN. Therefore, we have developed an in‐house water equivalent phantom capable of performing in any of axial, sagittal and coronal planes measurements for film coupled with point dose measurements. The film size used is 20.3 × 25.4 cm^2^, which enables a much larger measurements area when compared to StereoPHAN. This PSQA tool enhances the versatility for vertebra SBRT PSQA, allowing for a more comprehensive evaluation of dose distributions in scenarios where larger lesions or axial measurements are deemed valuable. The future work includes publishing further information in relation to this PSQA tool.

## 5 CONCLUSIONS

This study has compared the SRSMC to Gafchromic EBT 3 film for PSQA of vertebra SBRT, which is challenging due to the small fields used during delivery and high dose gradients between the target and organs at risk. The results showed a moderate negative correlation between EM and Γ for the SRSMC on the HD‐MLC system. However, there was no correlation between gamma pass rates of film and SRSMC. Both the SRSMC and film were able to accurately measure the high dose gradient in vertebra SBRT plans delivered by both MLC types. HD‐MLC plans possess a higher dose gradient with a smaller variation compared to M‐MLC plans. The gamma criteria and action and tolerance limits calculated in this study are more stringent than TG‐218′s suggested universal action and tolerance values. We propose the use of process‐based tolerance and action limits, including a 1 mm DTA. In this context, SRSMC emerges as a viable tool for vertebra SBRT PSQA.

## AUTHOR CONTRIBUTIONS

Kemal Berk conceived and designed the study, data acquisition, analyzed and interpreted the data, and wrote the manuscript. Nicholas Hardcastle provided expertise in detectors, contributed to data interpretation, and critically reviewed and edited the manuscript. Tomas Kron provided expertise in detectors, contributed to data interpretation, and critically reviewed and edited the manuscript. Adam Yeo contributed to the design of experiments, supervised the project, provided resources, contributed to data interpretation and critically reviewed and edited the manuscript.

## CONFLICT OF INTEREST STATEMENT

The authors declare no conflicts of interest.
